# Rectal Foreign Body: An Unusual Case of Self-Harm

**DOI:** 10.7759/cureus.69277

**Published:** 2024-09-12

**Authors:** Sima Patel, Ceri Gillett

**Affiliations:** 1 Surgery, New Cross Hospital, Wolverhampton, GBR

**Keywords:** deliberate self harm, foreign bodies, general surgery, rectal foreign body, urgent laparotomy

## Abstract

Although becoming more common, a foreign body in the rectum remains a challenging presentation. This is due to both a lack of clarity of treatment algorithms and established clinical guideline pathways and trying to avoid invasive methods to improve patient outcomes. We present a case where a foreign body was inserted into the rectum as a form of self-harm requiring emergency theater in the form of a sigmoid colectomy.

## Introduction

Although infrequent, the insertion of rectal foreign bodies is becoming an increasingly common presentation within the emergency department, particularly in urban areas [1-3]. The insertion of a foreign body via the rectum can be due to a multitude of reasons, including but not limited to diagnostic or therapeutic instrumentation, sexual gratification, assault, and accidental or drug concealment [2,3]. A presentation with no clear and agreed local pathway can prove difficult to manage, and although the utilisation of exploratory laparotomies is often avoided, there may be occasions where this is the only option. We present a case where the insertion of a foreign body per rectum was used as a form of self-harm and resulted in the patient undergoing a diagnostic laparotomy and sigmoid enterotomy.

## Case presentation

An 18-year-old male presented to the emergency department following the insertion of a lightbulb, per rectum, as a form of self-harm on a background of complex mental health needs. The patient presented within 12 hours following the insertion of the foreign body. On clinical examination, the abdomen was noted to be soft, with no features of peritonitis; on digital rectal examination, no blood was noted, and the glass of the bulb could be palpated. However, retrieval was not possible within the department.

**Figure 1:**
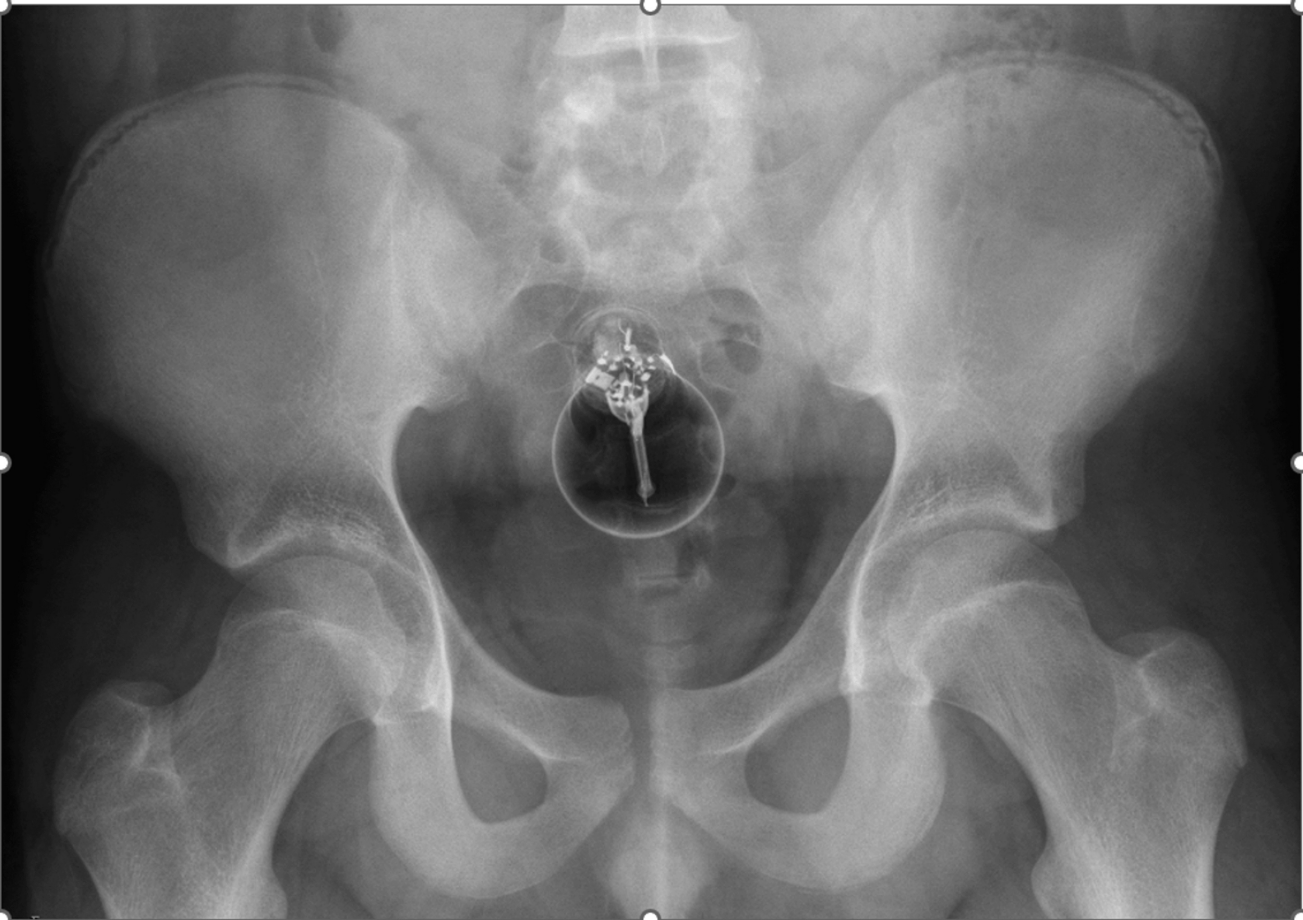
Anteroposterior X-ray of the pelvis showing the foreign body

The patient was admitted, and further imaging was performed in the form of abdominal and pelvis X-ray, with both anterior-posterior and lateral views to be able to appreciate the location of the lightbulb and to assess whether the lightbulb was intact. Figure 1 shows the anteroposterior X-ray of the pelvis which highlights the foreign body.

**Figure 2:**
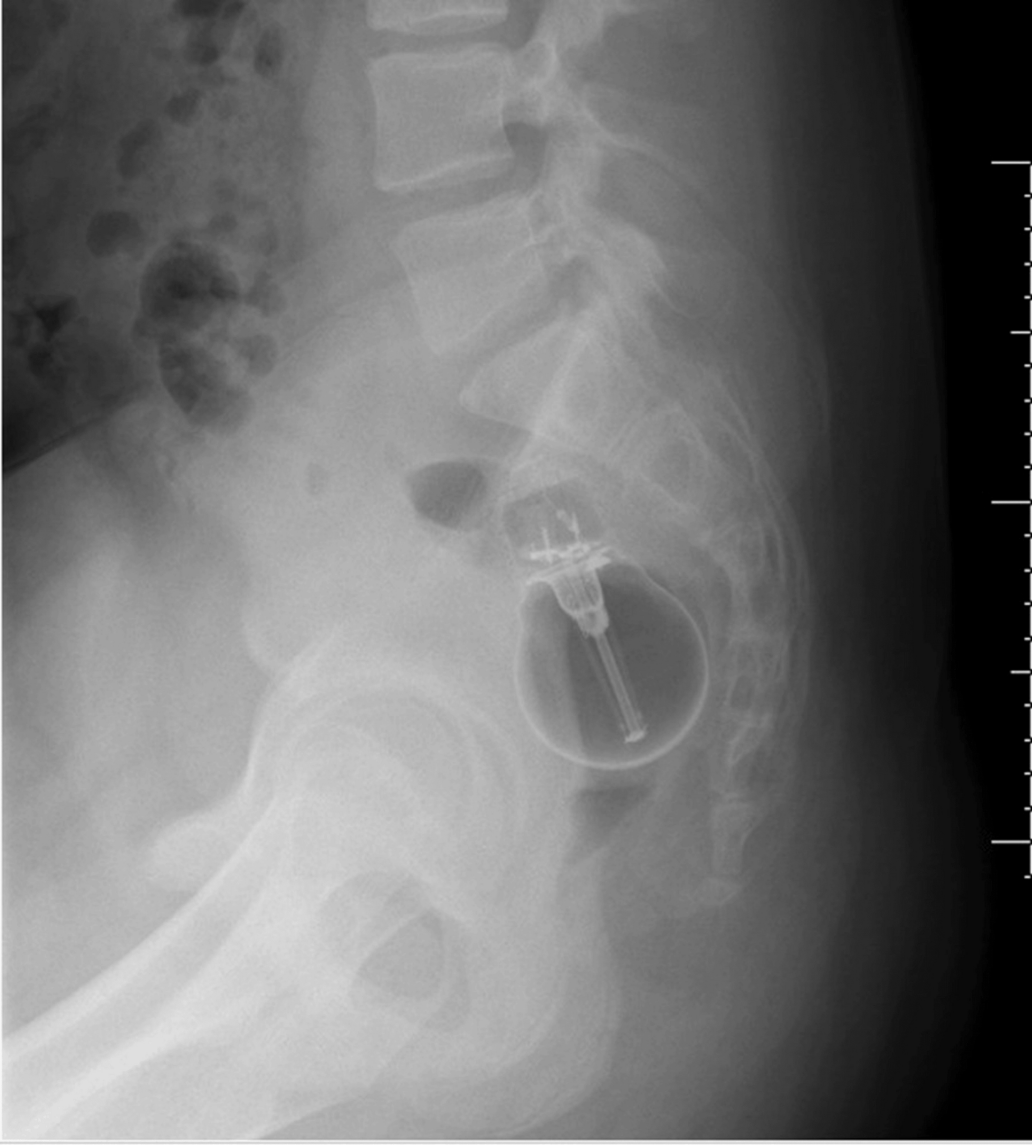
Lateral view X-ray of the pelvis

Figure 2 shows the lateral view of the foreign body. The lateral view was obtained to better appreciate the position of the item so to aid retrieval. 

The location of the foreign body was considered to be the proximal rectum; however, the glass nature of the lightbulb meant that a cautious approach needed to be adopted to avoid breaking the glass. Breaking the glass could lead to damage to both bowel and rectal sphincters with far-reaching consequences.

**Figure 3:**
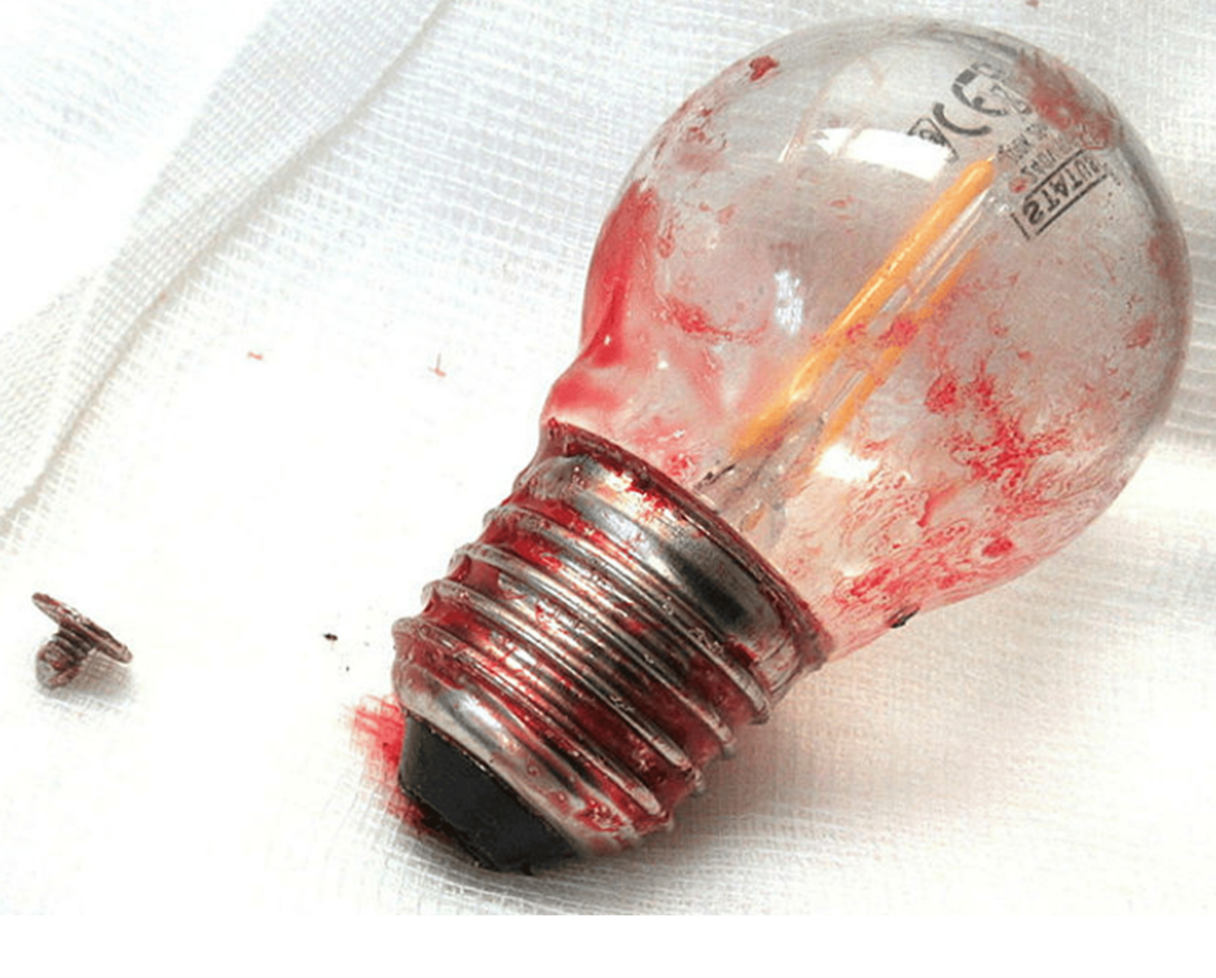
Lightbulb following retrieval

The patient was taken to the emergency theater, and a trans-anal approach was attempted with the patient placed in the Lloyd Davis position. However, the attempt was abandoned due to concerns regarding the glass breaking and then causing further injury due to the bulb being high up and firmly stuck in the proximal rectum. A horizonal incision was made in the left iliac fossa, and layers of the anterior abdominal wall were divided until the peritoneum was encountered and then divided horizontally to expose the sigmoid colon. Following manipulation of the lightbulb proximally into the pelvis, an enterotomy was performed in the sigmoid colon through a vertical incision, the lightbulb was then retrieved, and the colon was closed via interrupted sutures placed horizontally. Figure 3 shows the lightbulb following retrieval. 

The patient’s postoperative journey was uneventful, with flatus noted day 1 postsurgical intervention and bowels opened day 3 postsurgical intervention with no loss of anal tone noted. Discussion with the patient prior to discharge revealed that this was his attempt at self-harm, and so the patient was referred to the mental health support team for ongoing care. 

## Discussion

The insertion of a foreign body in the rectum is becoming a more common presentation to the emergency department, with the general surgical team asked to give input into these patients' care. Various objects have been described in the literature as being inserted into the rectum, ranging from fruit and vegetables to illicit drugs and batteries [4].

Presentation to the emergency department is usually following an unsuccessful attempt at self-retrieval of the object from the rectum, with both male and female patients presenting to the emergency department for further assistance. Patients may not be willing to divulge the truth when taking a history for fear of judgement or embarrassment; therefore, it is imperative that these patients are managed in a sensitive fashion to ensure no information is missed which may prove vital to management.

Within the United Kingdom (UK), the National Health Service (NHS) has a dataset which records activity occurring within the NHS. Between 2013 and 2023, 4,939 foreign bodies have been documented as having been removed from the rectum, with 348 bed days attributed to foreign bodies in the rectum between 2010 and 2019, with a cost of approximately £3,049,371 [5,6]. These figures rely on accurate coding of a procedure and so may not accurately reflect the true number which may be far higher [6,7].

Data from within the NHS suggests a greater male predisposition for rectal foreign bodies, with 81.7% of all presentations within the NHS being male between 2010 and 2023 [5]. This male predisposition appears to be similar to figures reported in international publications [4,6,8,9].

The insertion of any foreign body into the rectum can be associated with varying degrees of injury, from simple mucosal tears and lacerations leading to pain and bleeding, which may require no intervention, to perforation of the bowel, which may need more extensive surgical input [6,7]. Investigation of the rectal foreign body should involve clinical examinations, a digital rectal examination when safe to do so, X-rays to assess the object further, and consideration of a contrast-enhanced computed tomography for those with delayed presentation of over 24 hours or with whom perforation is being considered [3].

Kasotakis and his team suggested a workup and management algorithm for patients presenting with a foreign body in the rectum which uses features of peritonitis as a defining criteria as to when a patient would be managed via a laparotomy compared to a trans anal approach [4]. This algorithm allows a clinician to have a broad basis for this clinical judgement; however, the variability of objects inserted, the availability of resources and the surgeon’s clinical experience will mean that further work would be needed to develop a robust, evidence-based management algorithm that can be utilised within the NHS.

With a trans-anal approach being the least invasive, this option could be one that clinicians consider in the management of rectal foreign bodies. Following a thorough assessment, aided with appropriate imaging to ensure there is no danger of a sharp’s injury to the clinician, manual removal of the foreign body via the rectum can be considered in the stable patient with a low rectal foreign body [10-12].

Literature has described the use of local nerve blocks, spinal anaesthesia and general anaesthesia to help with the extraction of the foreign body to avoid laparotomies, and through providing suitable analgesia, local relaxation of sphincters and improve exposure of the foreign body to aid retrieval [9,4,13-15].

A colonoscopy can also be utilised for the removal of foreign bodies, although this would be user dependent and the qualities of the foreign body would determine if this would be a viable option, with glass or sharp objects being less suited due to the risk of further injury [12]. The use of endoscope snares to help move the foreign body down into the rectum have been described in literature, although following a review of 93 cases, it was determined that those objects in the sigmoid colon are more likely to require surgical intervention [15].

Failure of a trans-anal or an endoscopic approach, or if the patient presents with features of peritonitis, may warrant surgical intervention. Surgical intervention can range from laparoscopic-assisted retrieval of foreign body, laparotomy with an enterotomy, or symphysiotomy for objects that are wedged in the pelvis and cannot be retrieved even via an open laparotomy [4].

Following successful removal of the foreign body, post intervention monitoring is essential to ensure no further damage has occurred and the patient is medically fit for discharge. Post intervention monitoring can be in the form of serial abdominal examinations, monitoring bowel motions, and an abdominal X-ray to confirm removal of the foreign body and ensure no other injury. It is worth noting that where there is a concern regarding assault, appropriate support should be in place prior to discharge to allow ongoing support within the community [15].

## Conclusions

Early comprehensive assessments including consideration of patient factors, the object nature, size, shape, and location as well utilisation of imaging are all required in the successful management of rectal foreign bodies. A pragmatic approach should be sought, and although no set agreed evidence-based guidelines exist due to the variability in presentation and of objects inserted, the general consensus is that where a nonoperative approach is not possible or safe to attempt, exploratory laparotomy may become inevitable, with consideration of pubic symphysiotomy when objects are tightly wedged and cannot be removed even with open techniques.
